# The evolution and devolution of cognitive control: The costs of deliberation in a competitive world

**DOI:** 10.1038/srep11002

**Published:** 2015-06-16

**Authors:** Damon Tomlin, David G. Rand, Elliot A. Ludvig, Jonathan D. Cohen

**Affiliations:** 1 Department of Psychology University of Colorado, Colorado Springs Colorado Springs, CO 80918, USA; 2 Department of Psychology, Department of Economics, School of Management Yale University, New Haven, CT 06520, USA; 3 Princeton Neuroscience Institute Princeton University, Princeton, NJ 08540, USA; 4 Department of Psychology University of Warwick, Coventry, CV4 7AL, UK; 5 Department of Psychology Princeton University, Princeton, NJ 08540, USA

## Abstract

Dual-system theories of human cognition, under which fast automatic processes can complement or compete with slower deliberative processes, have not typically been incorporated into larger scale population models used in evolutionary biology, macroeconomics, or sociology. However, doing so may reveal important phenomena at the population level. Here, we introduce a novel model of the evolution of dual-system agents using a resource-consumption paradigm. By simulating agents with the capacity for both automatic and controlled processing, we illustrate how controlled processing may not always be selected over rigid, but rapid, automatic processing. Furthermore, even when controlled processing *is* advantageous, frequency-dependent effects may exist whereby the spread of control within the population undermines this advantage. As a result, the level of controlled processing in the population can oscillate persistently, or even go extinct in the long run. Our model illustrates how dual-system psychology can be incorporated into population-level evolutionary models, and how such a framework can be used to examine the dynamics of interaction between automatic and controlled processing that transpire over an evolutionary time scale.

Humans have a remarkable capacity for rational, deliberative thought. This can be especially important in considering the benefits of long-term goals and planning accordingly. Why is it, then, that we often appear to behave irrationally, succumbing to immediate temptations and acting against our own long-term interests? Part of the answer may be found in dual-system theories of cognition^1^,^2^,^3^,^4^,^5^,^6^. These theories posit two types of processes: automatic ones that produce rapid, stereotyped responses with little effort and controlled ones that are slower and more effortful, but also more flexible. Dual-system theories have received considerable support in psychology^7^,^8^,^9^,^10^, economics^11^,^12^,^13^ and neuroscience^14^,^15^. Understanding the interaction of these two systems may be essential for understanding important social and ecological problems that we confront in the modern world, such as shortfalls in retirement savings, increases in the prevalence of obesity and drug addiction, depletion of the environment, and other seemingly irrational behavior^16^,^17^. In this paper, we use an evolutionary model to begin to generate such an understanding.

Automatic processes can be viewed as responses optimized to specific internal or environmental signals^18^,^19^,^20^ that have emerged either over the course of evolution (e.g., “hardwired” reflexes) or as a consequence of extensive training (e.g., athletic skills or reading). Though these responses may be optimal within the context in which they developed, they are inflexible (i.e., context insensitive) and, if elicited under circumstances other than those in which they were developed (such as the current environment faced by most humans), may prove to be suboptimal^21^,^22^.

Intriguingly, perhaps the factor most responsible for the aforementioned change in the human environment is the emergence of the human capacity for controlled processing. Controlled processing (often referred to as “cognitive control” or “executive function”) and the flexibility it affords is fundamental to all of the faculties considered to be characteristically human — including reasoning, problem solving, language, and planning. These faculties, in turn, have given rise to an explosion of technologies that have dramatically changed our environment. However, we still carry with us those evolutionarily older automatic processes that may, in some circumstances, favor different behaviors than controlled processing (for more on the distinction between automatic and controlled processing, see Supplementary Text S1).

Why has the human brain preserved automatic responses when the ability to respond flexibly would seem to confer considerable adaptive advantage? Upon first inspection, it may seem that controlled processing should dominate automatic processing. While deliberation may be effortful and take time^3^,^7^,^23^, it has the considerable advantage of allowing the agent to respond effectively to its current circumstances and anticipate future ones. Thus, agents with the capacity for flexible responding should perform more favorably and, over the course of evolution, come to dominate the population. There are several reasons, however, why this may not be an inexorable outcome. For example, evolution toward exclusively controlled processing may be constrained by architectural factors that preserve structures underlying automatic processing^24^.

We propose an alternative: that interactions between controlled and automatic processing, and their interaction with the environment, may constrain the prevalence of controlled processing. More specifically, we consider the possibility that these interactions produce evolutionary dynamics in which (a) automatic processing is (or may become) more adaptive, or (b) that there is no stable equilibrium, but rather a continual oscillation in the dominance of the two types of processing. These dynamics may occur because the very success of controlled processing may lead to its downfall: the benefits that control allows may support either overgrowth of the population, or the invasion of agents that rely entirely on automatic processing, a type of “free loader” effect.

In the work presented here, we investigate the interplay between simplified forms of automatic and controlled processing in a particular setting: an environment in which agents in a population gather a resource (e.g., food) in order to survive and reproduce. We represent automatic and controlled processing in terms of two differing consumption policies: one that is rigid and has a strong immediacy bias (automatic), and the other that is flexible, and adapts to the environment in a future-oriented manner (controlled). While this domain — often referred to as intertemporal choice — is only one of many in which dual-system accounts of cognition are relevant, there are several reasons why it is an attractive starting point for studying interactions among dual-system agents at the population level.

First, it is a domain in which automatic and controlled processing make distinguishable contributions to behavior and there is growing evidence that automatic processing strongly discounts the future relative to controlled processing^11^,^13^,^15^,^25^,^26^. For example, interfering with controlled processing biases decision-makers toward options that are immediately gratifying but less remunerative^25^,^27^ or even possess negative consequences in the long term^28^. This is supported by evidence that the strong bias toward immediate rewards observed in human discounting behavior^29^,^30^,^31^ relies on evolutionarily older brain structures^7^,^14^, while future-oriented behavior relies on controlled processes that engage more recently evolved structures such as the prefrontal cortex^15^,^21^,^32^,^33^,^34^,^35^. Furthermore, theorists have provided formal arguments that the observation of preference reversals (e.g., choosing a deferred reward over an earlier but lesser one when both are in the future, but the lesser one as they draw closer in time^36^,^37^) and pre-commitment (choosing to make an immediately desirable resource unavailable so as to ensure the receipt of a more valuable one later^38^,^39^) can be rationally explained only in terms of competing systems of valuation^40^, a formal observation that is consistent with dual-system theories of cognition. Finally, intertemporal choice is a domain in which optimal performance can be precisely defined and operationalized in a relatively straightforward manner, thus permitting the design of agents that behave in a well specified and interpretable manner.

While there is growing consensus that controlled and automatic processing may compete within the individual to determine the outcome of intertemporal choice, there has been almost no work examining how agents with different biases may interact at the population level (though this question has begun to be examined in other domains^41^). For simplicity, consider a population in which there are two types of agents: one relying entirely on automatic processing (“automatic agents”) and another relying entirely on controlled processing (“controlled agents”). Assume that automatic agents do not have the flexible cognition necessary to consider the future, and thus exhibit a rigid policy with a strong immediacy bias^13^,^40^ – for example, they may consume all (or some fixed portion) of the resources that they encounter, irrespective of the availability of resources in the environment. In contrast, allow that controlled agents can deliberate, deciding how much of a resource to consume immediately, and how much to store for the future. The key distinction is *flexibility* – even if automatic agents did not immediately consume all resources but instead consumed them in some prescribed manner (such as genetically hardcoded manner in which squirrels save for the winter in a specific way), and even if this level could evolve over generations to adapt to new environments, control would still have an advantage because of the agent’s ability to adapt its behavior to current circumstances (e.g., current level of sustenance, estimate of the likelihood of finding more resources, and the amount of resource it has already stored). That is, the flexibility of controlled processing confers the ability to adapt behavior on an individual timescale, rather than an evolutionary one.

However, suppose that such flexibility comes at a cost, for example the extra time it takes to deliberate as compared to the implementation of a fixed and fast consumption policy^7^. Indeed, speed of processing is one of the defining features of automaticity^3^,^5^ and may provide an advantage to automatic agents in some contexts. For example, if an automatic must compete to acquire a good (e.g., with a controlled agent), use of a predefined policy may allow the automatic agent to act faster to consume the resource — while controlled agents stop to think about what would be optimal. We point to speed as one advantage of automatic processing, but there may be others — for example, efficiency (i.e., automatic behaviors may require less energy) and reliability. This tension between controlled and automatic processing may be a general property of intelligent systems — for example, it parallels a similar tension in computational systems between interpreted procedures (such as high level programming languages, that are flexible and adaptable), and compiled procedures (such as drivers, which are fast but limited in scope). Each has its advantages. What is less clear is how agents with these differing attributes interact.

Would the speed of automaticity outweigh the flexibility of controlled processing? How would factors such as the prevalence of resources or other agents affect the relative advantage of each policy and competition at the population level? How might secondary effects of controlled processing (such as increasing population size or the availability of resources) interact with these factors, and what would be the dynamics of these interactions? The answers to these questions are not obvious. Here, we introduce a theoretical framework for examining the dynamics of interactions among dual-process agents at the evolutionary timescale, and apply it to shed light on these questions.

## Methods

### Population model

In the conceptual example above, there were two distinct types of agents: automatic and controlled. In our simulations, we considered a more realistic case involving actual *dual*-process agents that used both kinds of processing: each agent had some probability of overriding its automatic response using controlled processing. For simplicity, the model treated this distinction between automatic and controlled processing as dichotomous, holding aside the issues that cognitive processes may exist along a continuum^42^ and may even be relative: that is, a process may be “automatic” or “controlled” depending on the process(es) with which it competes^1^. Also for simplicity, we treated the choice between automatic and controlled processing at each time point as based on a fixed probability, holding aside contextual and strategic factors that might influence this decision. We return to a consideration of these interesting and potentially important issues in the Discussion. Agents encountered resources over time, chose how to consume versus store those resources, and competed with one another for access when they encountered the same resource simultaneously.

### Energy and fitness

We examined how the probability of using automatic versus controlled processing evolved in a population of dual-system agents as they encountered and consumed resources. Agents inhabited an environment in which they had a specified probability of finding a resource in each time period, and a specified probability of encountering another agent in each time step (we varied the number of agents to study the effects of competition; see Supplementary Text S2 for additional details). Each agent had an *energy level E* that varied over time and directly determined reproductive success (fitness). On each time step, *E* was decreased by a constant amount, representing the energy required to subsist. To offset this drain on their stored energy, agents consumed a single type of resource that was encountered on each time step with fixed probability *R* (*environmental “richness”*). When acquired, resources could either be consumed to increase *E* or, when acting in a controlled manner, stored to increase the agent’s reserve of resources, *S* (see below), for use in the future. Consuming resources increased *E* with diminishing effect, implemented as a concave utility function whereby consumption produced the greatest increases in *E* when it was lowest and lesser increases as *E* increased (see Supplementary Text S3 and Figure S1). Thus, agents implemented three important biological constraints: 1) living organisms continuously expend energy for survival (drain), 2) consuming exogenous resources yields energy (resource-dependence), and 3) consumption is associated with satiety (diminishing returns).

### Dual-process agents

We implemented the distinction between automatic and controlled processing in two simple ways, intended to capture the most fundamental features of this distinction. First, automatic processing always resulted in the full consumption of an encountered resource, while controlled processing was more flexible, using a consumption policy that was sensitive to current environmental conditions and the future value of the resource. (We also performed simulations in which the automatic policy changed over evolutionary time, adapting a fixed level of consumption from generation to generation; see Supplementary Text S8). Second, when an agent acting automatically and an agent acting in a controlled fashion encountered a resource at the same time, the agent using automatic processing acquired the resource (and the controlled agent got nothing), reflecting the greater speed (or efficiency) of automatic processing. We chose this implementation so as to strike a balance between capturing the most important and widely agreed-upon features of automatic and controlled processing, while keeping the model from becoming excessively complex^3^,^4^,^5^,^9^,^23^. For example, a more complex implementation of controlled processing might take account of the nature of an encounter (e.g., whether the competitor is more likely to act in an automatic or controlled manner). While such considerations are clearly an important direction for future research (as we consider in the Discussion), even the simple model we describe exhibits complex dynamics, and understanding these may provide a valuable foundation for the examination of more complex models in the future.

**Figure 1:**
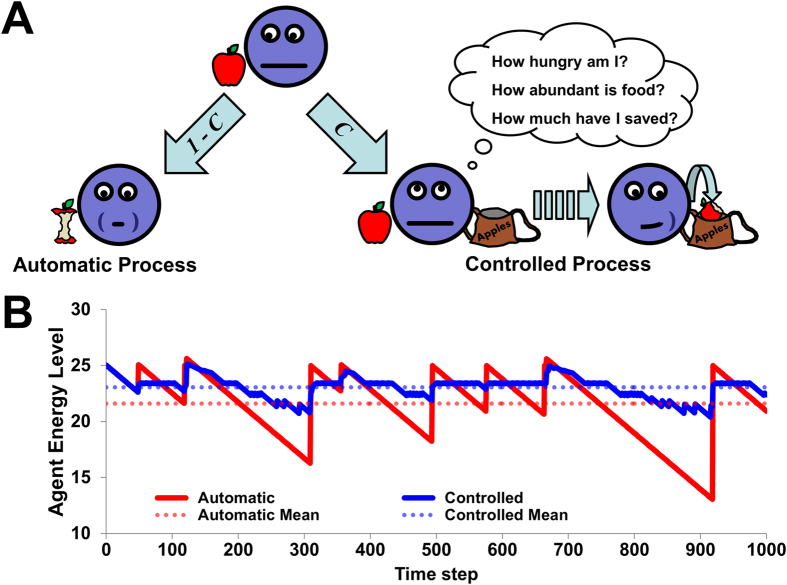
Consumption behavior and performance for automatic and controlled processing. (**A**) An automatic agent immediately and fully consumes any resource it encounters with probability 1 - *C*. A controlled agent uses its current energy level, how much it has stored, and the likelihood of finding resources, to determine the optimal amount to consume now versus store for later consumption (with probability *C*). However, controlled agents lose resources to the faster automatic agents when they are present simultaneously. (**B**) Sample energy levels over time for two agents: one using only automatic processing (red line), and the other using only controlled processing (blue line). The automatic agent experiences large, transient increases to its energy level, while the controlled agent maintains a higher mean energy level by storing resources and using them more efficiently. Energy levels are shown relative to worst possible mean performance (no consumed resources over the entire simulation).

We assumed that automatic processing was the default behavior, but that each agent had a stable probability *C* of over-riding this default and acting in a controlled manner. Thus, in a given time step, an agent acted in either a controlled or automatic manner (probabilities *C* and 1 – *C*, respectively) regardless of whether the agent encountered a resource or had to compete for that resource with another agent. If the agent acted automatically and encountered a resource, it consumed the entire resource and its *energy level E* was updated accordingly (Fig. 1A). If it did not encounter a resource, its energy level was simply decremented by the energy drain. In contrast, if the agent acted in a controlled manner and encountered a resource, it estimated an optimal amount *x* to consume from the resources available (the resource it just encountered plus those it had already stored, if any) through a process described below. By consuming the amount *x*, the agent increased *E* according to the concave utility function. Any unconsumed resources were stored, and the value of the agent’s energy reserve, *S*, was updated accordingly. If the agent acted in a controlled manner but did not encounter a resource, its energy level was decremented by the energy drain, the agent then chose an amount *x* to consume from its current store, and *S* was adjusted accordingly. Because agents acting automatically could not access the store, controlled processing in our model involved an idealized form of pre-commitment, in which stored resources could be accessed online in an optimal way and were unavailable when using automatic processing (for a relaxation of such constraints on automatic processing, see Supplementary Text S8, Figure S2 and Figure S3).

### Competition over resources

As we discuss in greater detail below, controlled behavior was optimal with respect to consumption. As noted above, however, this came at a cost: in direct competition for a resource, agents acting in a controlled manner lost to those acting automatically. In such instances, the automatic agent fully consumed the resource, while the agent using controlled processing got nothing but could still consume previously stored resources (in an alternate version of the model, we varied the probability of the outcome of the competition; as long as the probability that the automatic agent won was above 0.5, the results were qualitatively similar to those described here). If both agents used the same type of processing, then the resource was allocated randomly to one of them, and they applied their respective consumption policies. The probability of two agents simultaneously encountering a resource (and therefore competing over it) in a given time step increased with the population size (see Supplementary Text S2 for details).

### Optimal consumption under controlled processing

Because consumption was subject to saturation, the benefit from each additional unit consumed diminished as an agent’s *energy level E* increased. Optimal consumption therefore demanded that the benefit of consumption be weighed against the future value of the resource if it were stored and consumed later. If the probability of acquiring resources was low (i.e., the resource was scarce and/or competition was high), it would be wiser to save some of the resource for the future, when *E* was likely to be lower and the benefit of consumption higher. Optimal consumption thus depended on the current value of *E*, an estimate of the future availability of resources in the environment, and the amount of resources already stored.

The estimate of resource availability was experience-based, and thus sensitive to the effect of competition (because resources collected by other agents were not a part of the estimating agent’s experience). When agents used controlled processing, they took these considerations into account and balanced immediate consumption with storage so as to maximize *E* and thus fitness (see Supplementary Text S4 for details regarding this calculation).

Figure 1B depicts the energy levels of two different agents over time: one agent that always behaved automatically (red line) and another that always used controlled processing (blue line), each encountering an identical sequence of resources. The controlled agent exhibited both less volatility and a consistently higher mean *E* by storing an appropriate portion of its resources for later consumption. However, this superiority depended on the size of the population and the nature of the environment.

### Computation of agent fitness

Given the framework described above, an agent’s fitness was determined by 1) its level of control, 2) the probability that the agent would have access to a resource when it was in an automatic state, and 3) the probability that the agent would have access to a resource when it was in a controlled state. These latter two probabilities can be readily calculated from the *environmental richness R*, the *population size N*, and the distribution of *controlled processing C* across those agents. The two probabilities, weighted by the agent’s own value of *C*, determined the final probability that the agent would acquire a resource on any single time step. In each simulation, agents were given access to resources stochastically according to this single probability; for time steps on which the agent acquired a resource, the agent’s *energy level E* was updated according to its consumption policy (which was determined according to the probability *C* on each time step), and the next time step began. Prior to the evolutionary simulations (see below) we precomputed agent fitness, defined as mean energy level *E*, for a uniform sample of the possible values of the three determinants of agent fitness listed above through 30,000 simulations of 1,000 time steps each. In the evolutionary simulations, *R*, *N* and *C* were used to index this precomputed table of fitnesses, and the corresponding mean E was used for the agent’s fitness (see Supplementary Text S5 for details regarding these calculations).

### Evolutionary simulations

The population of *N* agents, each of which had its own value of *controlled processing C*, evolved according to the Wright-Fisher process^43^. In each generation, the population was reconstituted by sampling proportional to fitness from the previous generation, with replacement. The simulations included local mutation: with 5% probability, each new agent mutated, randomly adding or subtracting 0.02 from the value of *C* inherited from its parent (with *C* constrained to be between 0 and 1). Because we were interested in the spread of controlled processing within the population, simulations were always initialized with a homogeneous population of agents, each with *C* = 0. Thus, for each population of *N* agents in an environment with richness *R*, we computed distributions of control levels as they evolved across generations.

### Interaction between population size N, environmental richness R, and controlled processing C

We first examined how a fixed *population size N* and *environmental richness R* interacted with the distribution of values for *controlled processing C* in the population to influence the evolution of *C*. Together, *N* and *R* determined the likelihood of competition between agents over resources, which increased for higher values of both (see Results). The goal of later simulations was to characterize the dynamics in scenarios in which either *N* or *R* varied as a function of *E* and the propensity for controlled processing *C*. In *variable population size* simulations, *R* was held constant while increases in the mean *E* of the population increased *N*. In *variable richness* simulations, *N* was held constant while increases in the mean *E* of the population, coupled with higher frequencies of controlled processing, increased *R*. The latter explored the idea that surplus energy can be exploited by controlled processing (e.g., via technological innovation) to produce surplus resources.

### Variable population size simulations

Here, we considered the effect of population growth by allowing *population size N* to positively co-vary with population fitness. The population was initialized with a nominal population size *N*_*0*_. In each subsequent generation, if the mean *energy level E* of the population was above a specified threshold *T*_*N*_ by some margin (i.e., above *T*_*N*_ + ε), then *N* increased by a small fixed number of individuals, whereas if the mean *E* was below *T*_*N*_ by the same margin (i.e., below *T*_*N*_ – ε), *N* decreased by that number of individuals. If the mean *E* was between *T*_*N*_ – ε and *T*_*N*_ + ε, *N* remained constant, thus allowing for a population of stable size (rather than one that continually oscillated between two sizes). Thus, the threshold *T*_*N*_ parameterized the difficulty of maintaining a population’s size, and simulations explored the effects of different values of *T*_*N*_ and initial population size *N*_*0*_ (see Supplementary Text S6 for additional details).

### Variable richness simulations

Here, we considered the effect of innovation resulting from the interaction between surplus fitness (i.e., energy in excess of that needed for survival) and controlled processing (which could exploit surplus fitness to enrich the environment). For these simulations, the environment was initialized with nominal richness *R*_*0*_, representing the rate at which the environment yielded resources in the absence of innovation. In each generation, if the product of the population’s mean *energy level E* and its mean value for *controlled processing C* was above a specified threshold *T*_*R*_ + ε, *environmental richness R* increased by a fixed amount. This method therefore represented a simple version of the above concept – that innovations (such as environmental improvements) require the combination of deliberation and energy in order to be conceived and implemented. If E*C was below *T*_*R*_ – ε, *R* decreased by the same amount, but it was never allowed to fall below *R*_*0*_ (i.e., the baseline richness of the environment). If E*C was between *T*_*R*_ – ε and *T*_*R*_ + ε, *R* remained constant, thereby allowing for a population with a stable level of environmental richness. Thus, the threshold *T*_*R*_ parameterized the difficulty of modifying the environment, and simulations explored the effects of different values for *T*_*R*_ and *R*_*0*_ (see Supplementary Text S7 for additional details).

## Results

### Evolutionary outcomes for fixed combinations of population size N and environmental richness R

**Figure 2:**
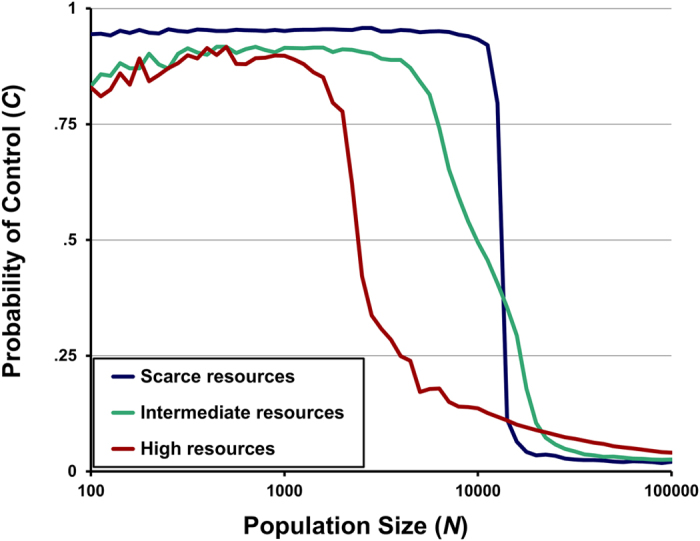
Equilibria depend on both *population size N* and *environmental richness R*. The x-axis represents the *population* size *N*, while the y-axis represents the propensity for *controlled processing C*. Each line corresponds to a different value of *environmental richness R*, with each data point indicating the mean value of *C* at the end of 100,000 generations for a given combination of *N* and *R.* Values of *R* were .005, .025, and .125 for “scarce,” “intermediate,” and “high” resources, respectively.

Figure 2 shows the results of initial simulations in which *population size N* and *environmental richness R* had fixed values. Higher values of *N* favored automatic processing because a larger *N* yielded a higher probability of competition (in which case automatic agents won the resource). Higher values of *R* also favored automatic processing because greater resource availability decreased the benefit of storing resources for future consumption.

These simulations also showed that *N* and *R* interacted to determine the success of controlled versus automatic processing: lower levels of *R* were associated with a greater range of *N* for which controlled processing was an equilibrium. Importantly, at higher values of *R*, there was also a range of values for *N* at which equilibria were reached with an intermediate value of *controlled processing C —* that is, in which dual-processing was a stable strategy, with agents sometimes acting automatically and sometimes acting in a controlled fashion (with a fixed probability).

### Variable population size simulations

**Figure 3:**
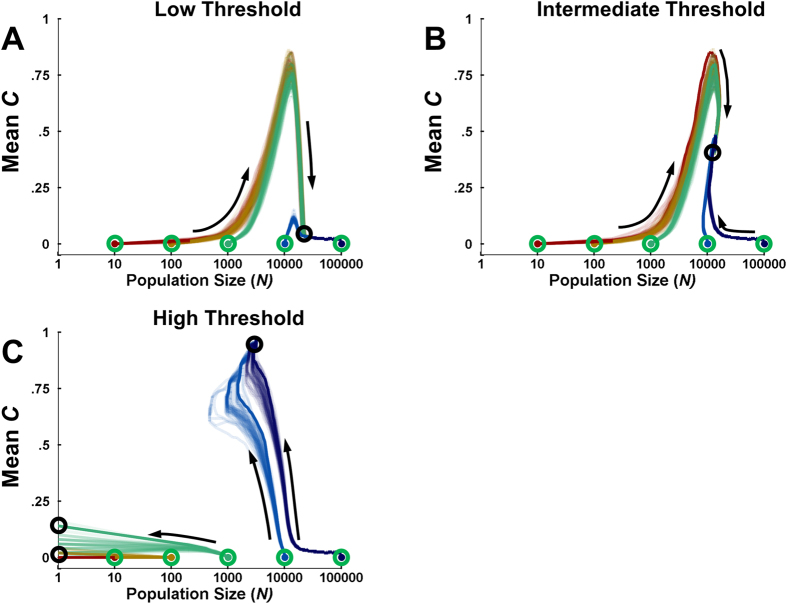
Variable population size simulations. Each panel shows evolutionary trajectories, starting with a value of zero for *controlled processing C* but with incrementally larger initial population sizes (x-axis). For each set of trajectories, a single exemplar is shown with a dark line, with dark circles indicating the final disposition of these exemplars. **(A)** When the threshold *T*_*N*_ was small and maintaining *population size N* was relatively easy, small populations evolved controlled processing until the increased population size made automatic processing more advantageous. (**B**) For an intermediate value of *T*_*N*_, populations reached a stable size only after evolving a moderate degree of *C.* (**C**) For high values of *T*_*N*_, populations required a very high fitness in order to maintain their current size, and only those that begin with high *N* evolved sufficient control to generate the needed fitness; smaller populations died out before they could do so. For all panels, the *environmental richness R* = .005.

Figure 3 shows the results of simulations in which *population size N* varied across generations based on the population’s fitness (mean *energy level E*). When the threshold *T*_*N*_ was low (Fig. 3A), relatively little fitness was required for the population to grow. If the population started with a small value of *N*, it began to evolve a high level of controlled processing due to limited competition. This in turn increased fitness, which increased the size of the population. However, as the population grew, the increased pressure due to competition led to the reemergence of automatic processing and diminution of control. Populations that started with a large *N* reached the same equilibrium, but the high levels of competition were already present and controlled processing did not become prevalent at any point.

With intermediate *T*_*N*_, it was more difficult to sustain a large population (Fig. 3B). In this case, small and large starting populations alike reached an equilibrium characterized by a moderate degree of controlled processing (for small starting populations, this equilibrium was achieved only after *controlled processing C* peaked and then diminished).

When *T*_*N*_ was high, it was difficult for populations to achieve sufficient mean fitness to grow, or even maintain their size (Fig. 3C). Only the efficient resource consumption conferred by controlled processing could yield such a high mean fitness. As a consequence, small starting populations died out before they could evolve a sufficient prevalence of controlled processing. Larger populations, however, could sustain greater losses before dying out, providing enough time for controlled processing to evolve and go to fixation. Accordingly, larger populations evolved controlled processing quickly enough not only to halt the shrinking of *population size N*, but to increase *N* once they approached an equilibrium value near *C* *=* *1*. Although the aforementioned simulations suggest that a large population of entirely automatic agents could not grow to such a size in the simulated environment, this does not preclude the invasion of that environment by one or more such populations. Thus, we see that when population size increases with population fitness, a boom-bust pattern can occur in which controlled processing initially invades and grows the population, but then is overwhelmed by automatic processing as competition becomes too high.

### Variable environmental richness simulations

**Figure 4:**
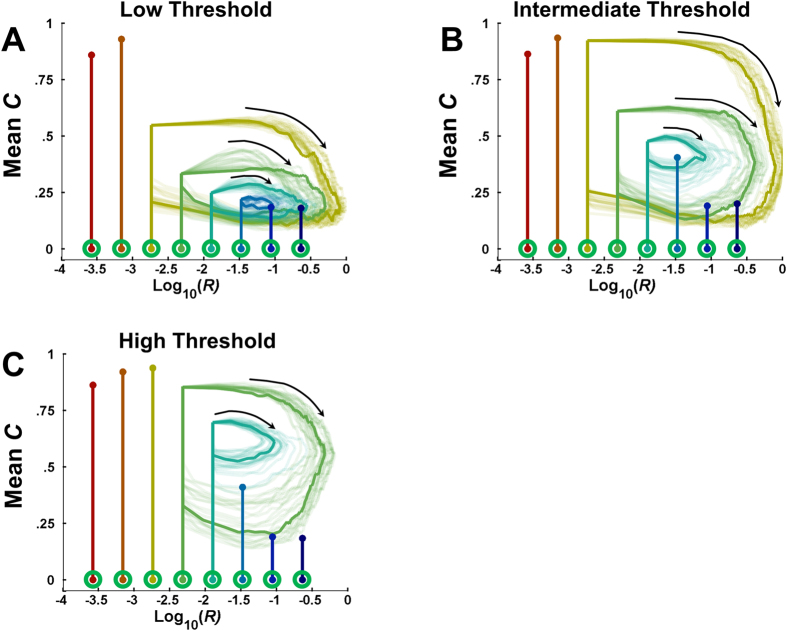
Variable richness simulations. The lines in each panel shows evolutionary trajectories, starting with *controlled processing C* = 0 but with incrementally higher values of *R*_*o*_ (x-axis). For those populations that exhibit limit cycles, many cycles are depicted and a single exemplar is shown with a darker line. *Population size N* was fixed at 10,000 for all simulations. (**A**) When the performance threshold *T*_*R*_ was small and several populations were able to modify *environmental richness R*. (**B**) A larger *T*_*R*_ reduced the number of populations able to modify *R*, but increased the range of the resulting limit cycles. (**C**) When *T*_*R*_ was large, only two populations achieved sufficient *mean energy levels E* and mean *C* to modify *R*.

Figure 4 shows the results of simulations in which *R* varied according to the combination of the population’s mean *energy level E* and the prevalence of levels of *controlled processing C*. In lean environments where the initial richness *R*_*0*_ was low (left side of each panel), even populations that evolved controlled processing lacked the fitness necessary to enrich the environment. Conversely, when *R*_*0*_ was high (right side of panels), there was insufficient competitive advantage to controlled processing for it to evolve, and so there was little change to the environment.

For intermediate values of *R*_*0*_ (middle sections of each panel), dynamics arose that were more interesting and complex. Initially, *C* increased, in turn increasing *E*. Once E*C was sufficiently large*, R* also began to increase. But as *R* grew, controlled processing became less beneficial, thus allowing automatic processing to proliferate and *R* to decline. The system returned to a leaner environment with its nominal richness *R*_*0*_, which again favored controlled processing, restarting the cycle. This effect was relatively robust to *T*_*R*_, exhibiting limit cycles for intermediate values of *R*_*0*_ at three different levels of *T*_*R*_ (Fig. 4A–C).

## Discussion

We have presented a simulation-based evolutionary model in which agents used either of two types of processes to decide how to consume finite resources. Automatic processes were faster, providing an advantage when directly competing with other agents over a resource, but they followed a rigid consumption policy and thus fared poorly in leaner environments. In contrast, controlled processes were flexible, anticipating future needs and performing well in leaner environments. Nevertheless, when the advantages of controlled processing to the individual also had external consequences, such as increasing population size or resource abundance, these effects could diminish and even reverse the spread of controlled processing through the population. We used two different scenarios to show that the success of controlled processing can undermine its own prevalence, leading to the re-invasion of automatic processing and/or limit cycles in the prevalence of processing style of an evolutionary timescale. These non-linear dynamics do not reflect simply a property of either automatic or controlled processing, but rather a complex interaction between the consequences of types of processing and the environment. Furthermore, these effects existed over a range of values for the parameters implemented in our simulations, and thus did not depend critically upon the conditions of any single set of initial conditions.

In one scenario, we assumed that higher mean fitness increased the size of the population, while lower mean fitness caused the population to shrink. When maintaining the population’s size was relatively difficult (Fig. 3C), populations either died out or evolved sufficient levels of control to sustain themselves. When increasing the population’s size was relatively easy, the excess fitness generated by controlled processing allowed the population to grow. However, the increase in competition at large population sizes led to the diminution (Fig. 3B) or demise (Fig. 3A) of control. Thus, while controlled processing may be favored to become prevalent, it may also be self-limiting due to its externalities and downstream consequences.

In a second scenario, controlled processing enriched the environment. These simulations yielded dynamics that were also non-monotonic, and somewhat more complex, reflecting the interaction between types of processing, competition, and resource availability. In this case, limits to the evolutionary success of controlled processing were due to free-loader-like effects: enrichment of the environment made it possible for agents using automatic processing to flourish and eventually outcompete those using controlled processing, at least for a time, by taking advantage of the greater resource availability provided by controlled agents (Fig. 4).

In our model, agents employing automatic processing used a simple, rigid policy: they completely consumed any resources they found. Moreover, automatic agents did not have access to stored goods. In additional simulations, we relaxed these assumptions, allowing agents using automatic processing to consume resources according to a parameter that evolved over generations in the same manner as the probability of controlled processing, *C*. This parameter dictated a “target energy level”: if the agent’s current energy level was below this target level, the agent would consume any resources currently available (including those previously stored) until the current energy level attained the target level (in our main simulations, this target energy level was fixed to the maximum level, such that automatic processing always dictated full consumption). In these additional simulations, the automatic processing policy could adapt on an evolutionary time scale to best match the *environmental richness R*. Even under this framework, the rise and fall of controlled processing still occurred during variable population size simulations (see Supplementary Figure S2) and limit cycles still occurred in variable richness simulations (see Supplementary Figure S3). Thus, our main results are not unique to the assumption that automaticity involves immediate full consumption or that it cannot evolve, but instead are driven by the more general phenomenon of controlled processing adjusting to the environment more quickly than automatic processing. It is worth noting that such adaptations in automatic processing would only apply for that environment, and could not keep pace with environmental change that exceeded the pace of evolution (including those produced by the externalities of control). In fact, adverse consequences could ensue if the downstream effects of controlled processing outpace these forces^21^,^44^, as suggested by existence of large limit cycles over in the variable richness simulations. Sufficient adaptation could also occur via cultural transmission, which is also cumulative but more rapid than genetic adaptation^45^. Future work should investigate this idea more directly by examining the interaction between types of processing and the pace of both evolution and environmental change.

Taken together, the non-monotonicity of outcomes we observed under very different conditions and mechanisms supports the idea that flexibility at the individual level, while optimal for an individual in isolation, may not be optimal at the population level when it must compete with more rigid but more efficient strategies. These observations may have important scientific and practical implications, insofar as the balance between automatic and controlled processing influences decision making behavior in domains such as diet, drug use, savings, and the consumption of natural resources — behaviors that have consequences for both the individual but also the population and the environment.

These results make two significant contributions to the study of dual-process models of cognition. First, our framework establishes the utility of population models for studying the evolutionary trajectories of cognitive processes. Second, our simple case of evolutionary competition between controlled and automatic processing serves as a benchmark for future work that could consider more sophisticated forms of control. Such work might include control strategies that respond to, or even anticipate, the social and environmental effects of controlled processing, thereby potentially stabilizing the population at higher levels of controlled processing (and correspondingly higher mean levels of fitness).

The models we describe are relatively simple in other respects as well. More detailed models could consider environments with non-uniform spatial structure^46^, in which agents could flexibly adapt to localized distributions of resources to produce “cultural topologies” of controlled vs. automatic processing. It may also be useful to explore cases in which controlled processing uses heuristics to speed processing when facing competition^47^, anticipates and exploits the vulnerabilities of automatic processing (e.g., the role of marketing in promoting obesity), explicitly recognizes the value of prosocial behavior (e.g., cooperation^41^), is subject to distortions caused by social circumstances^48^, or is constrained sociologically (e.g., affects only others that are also using controlled processing). However, such strategies may carry other costs (e.g., energetic).

A further simplifying assumption of our simulations was that the controlled agents had access to the optimal consumption policy. Computing this optimal policy used a method that almost certainly exceeds the capabilities of real world agents. However, searching the space of possible actions need not be so exhaustive: computational architectures have been proposed that can dramatically limit search and still closely approximate optimal policies^49^,^50^. Experiments have also shown that humans can effectively and unconsciously prune such a space with simple heuristics^51^.

It is worth noting that our simulations focused on a particular application of cognitive control: the problem of intertemporal choice (i.e., deciding between immediate and delayed consumption). There is a growing body of work suggesting that cognitive control plays an important role in such decisions^14^,^15^,^25^,^26^,^32^,^52^ and, though it is only one domain in which cognitive control operates^2^,^3^,^16^,^53^,^54^,^55^, it may be a particularly important one. Intertemporal choice is fundamental to self-control: the capacity to behave in accord with long-term objectives despite temptations that have immediate benefits but long-term costs^11^,^40^,^56^,^57^. Self-control, in turn, is at the heart of many social and environmental issues^58^. Formal theory indicates that any rational account of self-control must posit at least two processes, one of which represents the agent’s immediate interests and the other (the controlled process in our model) that serves its long-term interests^40^. Thus, models that address phenomena having to do with self-control at the population level must be composed of agents built on at least a dual- (and possibly multi-) process model of decision making. The models we have described provide initial examples of this approach. More work, however, is needed to characterize the behavior of such systems in a fuller and more precise way, to incorporate more complex forms of control, and to validate this theoretical work in an empirical setting.

In summary, we introduced simulations exploring the evolutionary consequences of dual-system models of cognitive function, in which automatic and controlled processes competed for expression. We demonstrated that cognitive control, despite its capacity for flexible behavior and higher overall fitness, can evolve along a non-monotonic course as a result of its external influences on the population and/or the environment. That is, its initial spread can produce conditions that undermine its further evolution, and in some cases bring about its collapse. This type of model may be useful for exploring more sophisticated forms of controlled processing, and more complex interactions between individual decision making behavior and social or ecological factors. There are multiple domains in which different styles of human cognition can impact our wellbeing and that of our environment, and the computational modeling of large populations that embody these cognitive strategies is potentially applicable to a number of the problems posed by the very success of controlled processing in our own species.

## Additional Information

**How to cite this article**: Tomlin, D. *et al.* The evolution and devolution of cognitive control: The costs of deliberation in a competitive world. *Sci. Rep.* **5**, 11002; doi: 10.1038/srep11002 (2015).

## Supplementary Material

Supplementary Information
