# Supplementary material for: The evolution and devolution of cognitive control: The costs of deliberation in a competitive world
Source: Sci Rep. 2015 Jun 16;5:11002. doi: 10.1038/srep11002 (PMC4468419; doi:10.1038/srep11002)
Supplement: Supplementary Information [file srep11002-s1.pdf]

Supplementary Information

The evolution and devolution of cognitive control: The costs of deliberation in a competitive world

Damon Tomlin, David G. Rand, Elliot A. Ludvig, and Jonathan D. Cohen

Text S1. Notes on dual-process theories………………………………………………...1
Text S2. Probability of competition…………………………………………………………2
Text S3. Initial conditions and agent energy function……………………………………..2
Text S4. Balancing immediate consumption and storage during controlled processing...3
Text S5. Computation of agent fitness……………………………………………………...4
Text S6. Procedure for updating N in variable population size simulations………………5
Text S7. Procedure for updating R in variable richness simulations……………………5
Text S8. Procedure for simulations with evolving automatic processing………………..5
References…………………………………………………………………………………….6
Figure S1: Agent utility function……………………………………………………………..7
Figure S2: Variable population size simulations for evolving automatic processing…….8
Figure S3: Variable richness simulations for evolving automatic processing…………….9

Text S1. Notes on dual-process theories.

Under a dual-process framework, the "automatic" process subserves functions from homeostatic regulation to emotional responses, while the "controlled" process subserves more deliberative processes, but in turn relies upon functions such as working memory. Within this conceptual framework, each system is a type of processing, rather than a set of integrated processes, and the distinction between them is analogous to that between compiled and interpreted procedures in computer science. The former are optimized for a particular purpose (e.g., drivers for a specific printer), while the latter are more flexible, but less efficient and slower, requiring extra code and time for execution.

***Text S2. Probability of competition*.**

The probability of an agent being co-localized with a resource and at least one other agent is:

$$P(compete) = R * \left(1 - \left(\frac{L-1}{L}\right)^{N-1}\right) \quad [S3]$$

where *L* is the number of possible locations (in this case, 10,000). The simulation takes into account the increased competition that arises when multiple agents coincide on the same location, modeling each such agent as having an identical, but statistically independent value of *C*. In our simulations, if *N* = 100 there was a ~1% chance of having to compete with other agents at any given site. With 1,000, 10,000, and 50,000 agents, the probability increased to 10%, 63%, and 99%, respectively. In the work presented, agents engaged in automatic processing always beat agents engaged in controlled processing when they competed for a resource. In one version of the model, we varied the probability that the "automatic agent" would beat the "controlled agent"; as long as the probability was above 0.5 the results were not qualitatively different from the aforementioned limiting case.

***Text S3. Initial conditions and agent energy function*.**

In all the simulations, agents began each simulation with an energy level of 25, the amount of a new resource (*g*) was 55, the energy level drain (*d*) was .05 units on each time step, and the maximum size of the store was 200. If an amount *x* is consumed on time step *t*, the agent's energy level *E* on time step t+1 is given by:

$$E(t+1) = \log\left(e^{E(t)} + x\right) - d \quad [S1]$$

Figure S1 depicts the benefit of consuming a single resource over a range of initial energy levels. While the exact values for these parameters, and the mathematical form of the energy function, were chosen for computational convenience, the constraints they impose on the agent are rooted in nature. However efficiently an organism can convert food into stored energy, it must expend additional energy in order to move its increased

mass around the environment. Thus, the consumption of food as the organism's store of energy increases conveys diminishing marginal returns to the organism.

Text S4. Balancing immediate consumption and storage during controlled processing.

We assume that every agent continuously estimates the probability of acquiring a resource from its own experience using a frequentist probability (the number of resources encountered divided by the number of time steps experienced). This calculation takes into account both the probability of encountering the resource in the environment as well as the likelihood of encountering other agents and successfully competing for the resource. When an agent uses controlled processing it takes this estimate into account, together with its current energy level and the amount of the resource it has already stored.

Given these three values, the optimal amount to consume in order to maintain the highest mean energy level can be computed using value iteration[1, 2], a form of dynamic programming. In value iteration, the optimal policy (best amount to consume) is found by repeatedly calculating the value, *V,* of every possible state, *s*. The value of each state is equal to the value of the best action *a* from that state. In our calculations, there were 60,000 total states (300 discretized energy levels x 200 store levels) and up to 201 actions (consume between 0 to 200 resources from the store). Because the likelihood of encountering a resource was independent of the agent's policy, each policy was computed separately for 100 possible values for the estimated *R* (spaced logarithmically from 0 to 1). The value of each state was calculated as the sum of the reward (energy) from the consumed amount for the best action (r_a), plus the value of all possible resultant states (s') weighted by their likelihoods ($p(s')$):

$$V_{k+1}(s) = max_a[r_a + \sum_{s'} p(s') \times \gamma V_k(s')\,] \qquad \text{[S2]}$$

where γ =.999 is a discount factor that weights imminent rewards more heavily than distant ones. The value of the discount factor corresponds to the optimal policy in an environment that lasts 1000 time steps, the length that was used in the evolutionary simulations (see below). For any given action *a*, there were two possible resultant

states—determined by whether a resource was found or not found. These likelihoods were determined by the richness, *R*, of the environment (as estimated by the agent based on its experience). Through this equation, on each iteration *k*, the value-iteration algorithm cycles through every possible energy, store, and consumption level and re-calculates the value of each state based on the most recent estimate for the value of the resultant state. This tying of the value of a state to the value of its successor means that, with each iteration, the values (and thus policy) get closer and closer to the optimal. The optimal policy was separately computed for each richness *R* and stored in a look-up table, which was used to generate agent behavior in the evolutionary simulations.

***Text S5. Computation of agent fitness*.**

The fitness of an agent depends on its probability of using control, *C*, and two properties of the (physical and social) environment: 1) the probability that the agent has access to a resource when it is in an automatic state, and 2) the probability that the agent has access to a resource when it is in a controlled state. These two probabilities, in turn, can be readily calculated from 3 environment parameters: the environmental richness *R*, the number of agents *N*, and the distribution of controlled processing *C* across those agents. To decrease the computation time for the evolutionary simulations, we created a fitness look-up table by discretizing the *C* strategy space (which ranged in linear increments from 0 to 1) and these two probabilities (which ranged in $\log_{10}$ increments from 0 to 1). We filled the look-up table by computing the fitness of an agent under each combination of *C* and the two probabilities, simulated through 30,000 runs of 1,000 time steps each. In the simulations, the agent was given access to resources stochastically according to the above probabilities. For time steps on which the agent acquired a resource, the agent's *E* was updated according to its consumption policy, and the next time step began. This pre-computed fitness was then used in the evolutionary simulations.

Text S6. Procedure for updating N in variable population size simulations.

In this scenario, we considered the effect of population growth by allowing *N* to positively co-vary with the population fitness. This was implemented by scaling *N* up by ten individuals for the next generation if $E > T + .1$, scaling *N* down by ten individuals if $E < T - .1$, and leaving *N* unchanged if $T - .1 \leq E \leq T + .1$. Here, *T* is a threshold that describes the difficulty (in terms of energetic cost) of maintaining the population. The buffer of .1 was employed so that population sizes could reach an equilibrium value, rather than continually oscillate between two values. In Figure 3, panels A, B, and C represent $T = 10$, 15, and 20, respectively.

***Text S7. Procedure for updating R in variable richness simulations*.**

Here, we considered the effect of innovation resulting from the combination of controlled processing and sufficient fitness. For these simulations, agents in the first generation were placed in an environment with nominal richness R_0. Each generation could then modify *R* for the next generation based upon its mean values for *E* and *C.* This was implemented by increasing *R* by $5*10^{-4}$ for the next generation if $E*C > T + .1$, decreasing *R* by $5*10^{-4}$ if $E*C < T - .1$, and leaving *R* unchanged if $T - .1 \leq E*C \leq T + .1$.The next generation's *R* could not fall below R_0, which represented the richness of the environment without modification (i.e., the population could not degrade the environment below its "natural" productivity). Here *T* is the minimum value of *E*C* required for creating resource-increasing technologies. The buffer of .1 was employed so that resource rates could reach an equilibrium value, rather than continually oscillate between two values. Although Figure 4 depicts trajectories for $T = 5$, 10, and 15 (panels A, B, and C, respectively), limit cycles also exist for higher values of *T* and different values for the increment in *R*.

***Text S8. Procedure for simulations with evolving automatic processing*.**

In order to test whether the effects we have described only exist when automatic processing has a constant policy of maximum consumption, we implemented new simulations in which agents' automatic processes evolved over generations. Procedures were identical to the original simulations except for the addition of a single parameter: a

“target energy level,” *L*, that dictated an agent’s behavior whenever it used automatic processing. Under this framework, when an agent acted automatically it had access to available resources at its current location and those previously stored, just as it would if it were acting in a controlled manner. From these resources, the agent consumed as much as was necessary to reach the target energy level *L*. Thus, automatic processing had access to the same resources as controlled processing, enjoyed its competitive advantage, and adapted to the environment on an evolutionary timescale, but was still not *flexible*. Procedures for variable population size and variable richness simulations were the same, excepting that agents could mutate in terms of either their level of control *C* or their target energy level *L*.

References

1. Bellman, R. *Dynamic programming* (Princeton University Press, Princeton, NJ, 1957).

2. Sutton, R. S., Barto, A. G. *Reinforcement learning: an introduction* (MIT Press, Cambridge, MA, 1998).

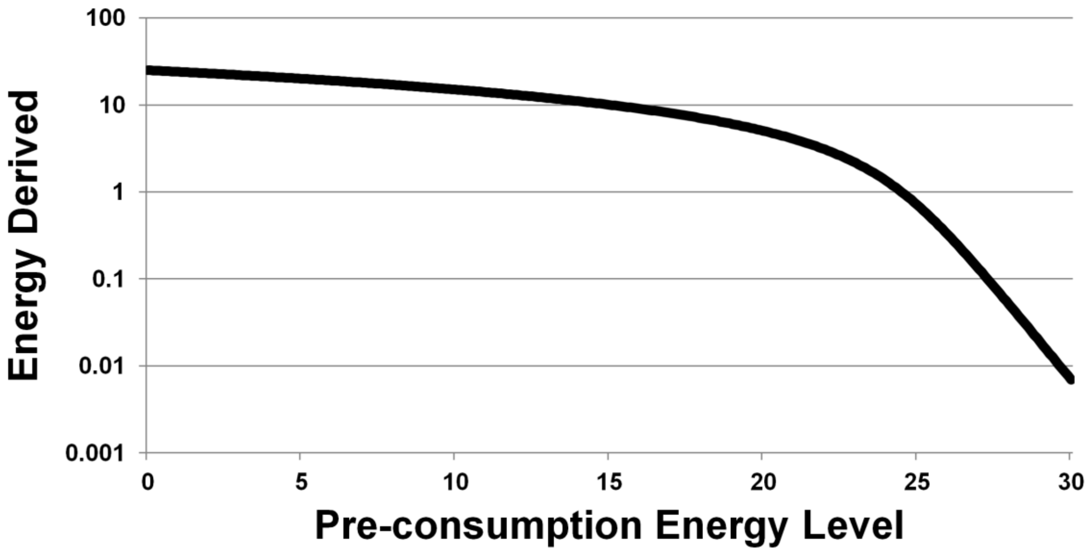

Fig. S1. Diminishing returns: the benefit of consuming a resource decreases as energy level increases. Because the agent's energy level increases logarithmically, the benefits an agent derives from a unit of resource is a decreasing function of its current energy level.

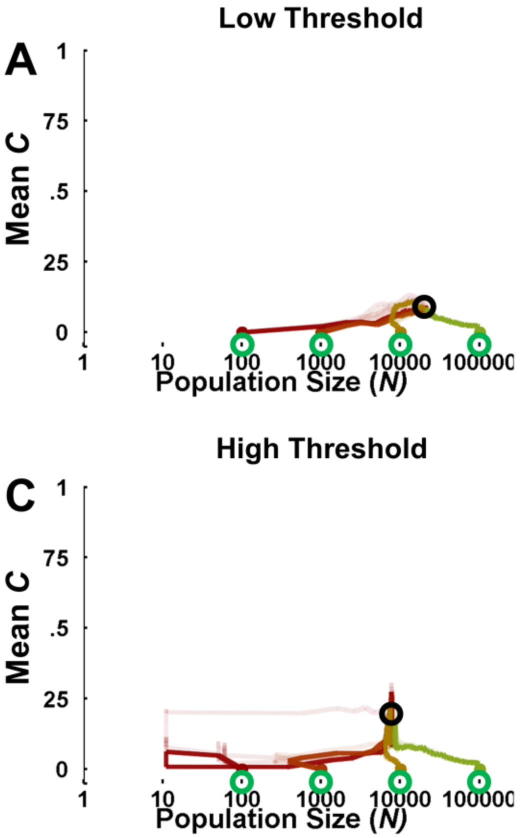

Figure S2. Variable population size simulations for agents whose automatic processes evolve across generations. Each panel shows evolutionary trajectories, starting with a value of zero for *controlled processing C* and *target energy level L* = 7 (effectively a policy of "consume everything"), but with incrementally larger initial population sizes (x-axis). For each set of trajectories, a single exemplar is shown with a dark line, with dark circles indicating the final disposition of these exemplars. Although controlled processing is generally less prevalent as a result of the evolution of automatic processing's policy, controlled processing still exhibits dynamics similar to those in Figure 3. **(A)** When the threshold T_N is small and maintaining population size is relatively easy, small populations evolve controlled processing until the increased population size makes automatic processing more advantageous. **(B)** For an intermediate value of T_N, populations reach a stable size lower than that for small T_N and evolve a slightly higher degree of *controlled processing C.* **(C)** For high values of T_N, stable population sizes are smaller and the necessary level of controlled processing is higher. For all panels, the *environmental richness R* = .02.

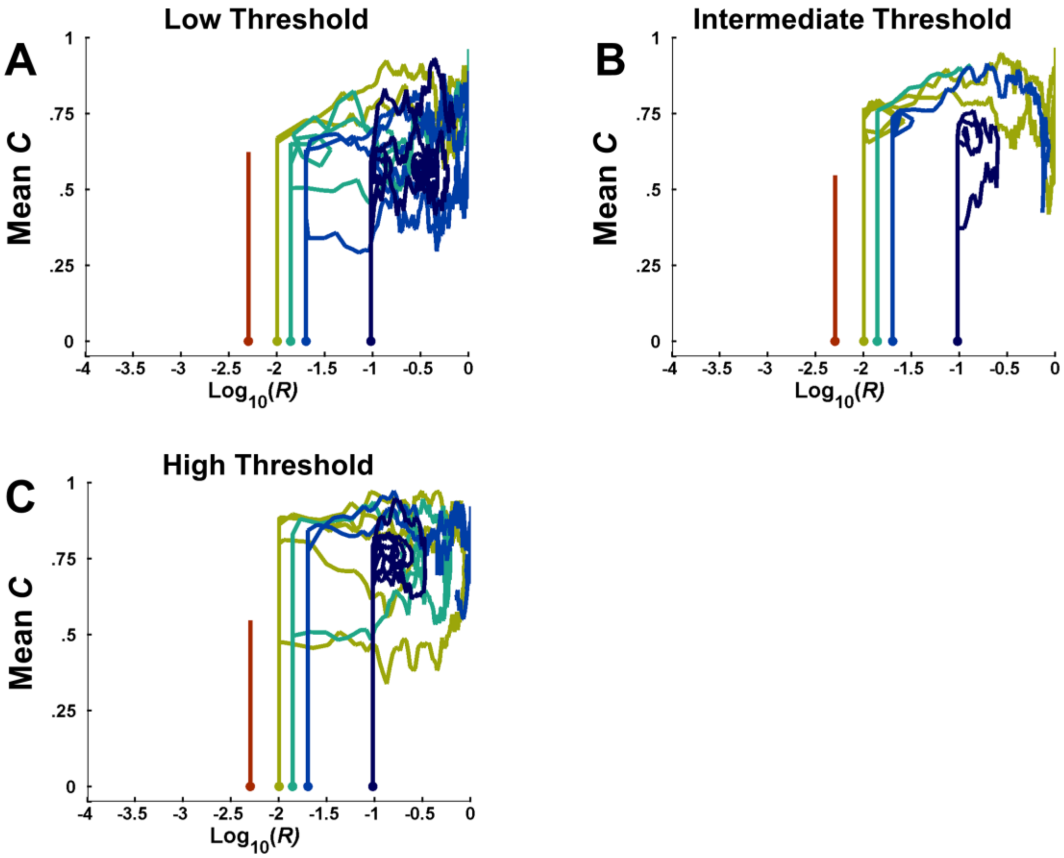

Figure S3. Variable richness simulations for agents whose automatic processes evolve across generations. The lines in each panel shows evolutionary trajectories, starting with *controlled processing* C = 0 and *target energy level* L = 7 (effectively a policy of "consume everything") but with different values of R_o (x-axis). *Population size* N was fixed at 10,000 for all simulations. As shown above, limit cycles similar to those in Figure 4 occur even when agents' policies when engaged in automatic processing are allowed to evolve. Cycles are depicted for low **(A)**, intermediate **(B)**, and high **(C)** values of the performance threshold T_R.
